# Evaluation of a pre-post quasi-experimental educational intervention on breast cancer awareness among pharmacy professionals in Karachi, Pakistan

**DOI:** 10.3389/fpubh.2024.1443249

**Published:** 2024-09-10

**Authors:** Noor us Saba Mansoor, Safila Naveed, Huma Ali, Ammara Manzoor, Saima Zahoor, Javeria Sheikh

**Affiliations:** ^1^Faculty of Pharmacy, Jinnah University for Women, Karachi, Pakistan; ^2^Faculty of Pharmacy, Karachi University, Karachi, Pakistan; ^3^Faculty of Pharmacy, Jinnah Sindh Medical University, Karachi, Pakistan; ^4^Department of Oncology, National Institute of Blood Diseases and Bone Marrow Transplant, Karachi, Pakistan; ^5^Department of Clinical Oncology, Jinnah Postgraduate Medical Centre, Karachi, Pakistan

**Keywords:** breast cancer awareness, educational intervention, pre- and post-study, quasi-experimental trials, breast self examination

## Abstract

**Introduction:**

Cancer, particularly breast cancer, is a major contributor to mortality and a significant impediment to life expectancy. In 2020, breast cancer accounted for 11.7% of all cancer cases and caused approximately 685,000 deaths worldwide, surpassing lung cancer in prevalence. The study aims to evaluate the impact of an educational intervention on breast cancer awareness among pharmacy students by comparing their understanding before and after the program.

**Method:**

A pre-post quasi-experimental study was designed to assess knowledge and awareness of breast cancer, breast self-examination (BSE) practices, and attitudes toward breast cancer among female university students in Karachi, Pakistan. Participants completed a pre-session questionnaire, attended an awareness workshop and video tutorial, and then completed a post-session questionnaire 2 weeks later.

**Results:**

Of 1,200 participants, 1,015 of them completed both the pre- and post-intervention questionnaires. Key demographic features included 83.9% of the participants being in the 18–24 age group, 26.8% being married, and only 14.2% having a family history of breast cancer. Before the intervention, 60.7% of the participants were not involved in regular breast self-exams due to a lack of awareness. Post-intervention results showed a significant increase in awareness, with 35.9% rising to 94.9%. The use of screening methods increased from 46.7 to 94.8%. Knowledge of breast cancer improved from 51.2 to 96.7%, and the general perception rose from 48.2 to 93.4%. Attitudes toward self-examination also shifted positively, indicating a significant change in perception.

**Interpretation and conclusion:**

The study concludes the baseline knowledge of breast cancer among female students was inadequate but improved significantly from over 40% to over 90% following the educational intervention. The intervention positively influenced the general perception and attitudes toward breast cancer. These findings highlight the need for regular educational sessions to enhance awareness, improve healthcare outcomes, and reduce mortality rates, particularly in developing countries.

## Introduction

Cancer remains a major contributor to global mortality, significantly impacting not only life expectancy but also the quality of life. While a notable decline in the mortality rates from coronary heart diseases and strokes has been observed, cancer continues to pose a significant threat. Among various cancer types, female breast cancer has emerged as a leading concern. In 2020, breast cancer surpassed lung cancer, accounting for 11.7% of all cancer cases globally. It also ranked as the ninth most common cause of death worldwide, with official reports documenting 685,000 deaths. Breast cancer is the most prevalent among women, affecting approximately 1 in 6 cancer-related deaths (159/185) and in 1 in 4 cancer cases globally (1).

In low-income countries, several factors contribute to the delayed diagnosis of breast cancer. These factors include limited resources, low awareness due to illiteracy, insufficient screening and inadequate treatment facilities, financial constraints, and cultural myths and hindrances. These obstacles prevent women from fully accessing available treatment options. Additionally, mobility restrictions further restrict their ability to seek appropriate healthcare services (2).

The most critical barrier to increasing awareness is the need for education. Adequate awareness of screening techniques, the importance of timely diagnosis, and proper clinical examinations can help women overcome structural and individual challenges, leading to more effective and cost-effective treatments.

Frequent breast cancer programs implemented in various local areas can significantly improve the alignment of clinically detectable diseases with timely and accurate diagnostic services, especially for women who show clear signs and symptoms of breast cancer. In developing countries, delayed diagnoses at advanced stages are common, with rates ranging from 30 to 50% in Latin America to 75% in Sub-Saharan Africa. These advanced stages are often self-detected by patients through symptoms such as lumps, watery discharge, nipple inversion, or other noticeable changes in their breasts (3).

A well-managed social security system is vital for providing effective healthcare to the lower class and alleviating economic and social constraints. Unfortunately, Pakistan lacks such a system, resulting in negative health outcomes for impoverished populations (4, 5). Despite growing awareness about breast cancer and an increasing demand for diagnostic and treatment services, several factors prevent Pakistani women from seeking breast examinations and treatment. These factors include the inaccessibility of tertiary care hospitals in rural areas, cultural norms, and a lack of awareness. Together, these issues discourage open discussions about breast and reproductive health. Research indicates that Muslim women often prefer female physicians for examinations of private body parts and pregnancy checkups, as this helps them avoid the hesitation and discomfort they might experience when dealing with male physicians (6, 7).

Additional factors such as age, marital status, dependency, social restrictions, and varying codes of honor further limit women’s access to timely healthcare and optimal treatment. Younger women, in particular, face greater challenges in accessing healthcare compared to older women (8). Social and family support plays a critical role in patients’ psychosocial and physical wellbeing. Unfortunately, in countries like Pakistan, these aspects are often undervalued, which leads to social isolation and depression, ultimately hindering the psychosocial and physical recovery of women (5).

The World Health Organization (WHO) recommends early diagnosis as the most effective approach to controlling breast cancer (3). Early diagnosis can be achieved through awareness of screening methods such as breast self-examinations (BSE), clinical breast examinations (CBE), and mammography. Assessing women’s awareness of these methods can help health decision-makers identify information gaps and improve timely diagnosis and treatment, ultimately reducing mortality rates (9).

Mammography is the leading screening method for breast cancer globally and has been associated with a reported 25% reduction in mortality rate among those aged between 50 and 69 years. However, it is less beneficial for women aged 40 to 49 years (10), especially in developing countries where population-based mammography is often unavailable due to funding and infrastructure limitations (11–13). Consequently, clinical breast examination and BSE are emphasized as alternative methods in regions lacking national mammography programs.

Bi-monthly BSE, as recommended by Haagensen, can help detect tumors early and reduce the incidence of advanced malignancies. CBE, which involves the palpitation and inspection of both breasts by medical professionals, has shown varying effectiveness in low- and middle-income countries. Given the diverse sociocultural norms and contextual factors, breast cancer screening strategies should be tailored to each country’s specific needs through customized workshops (14).

### Study objectives

The purpose of this research is to examine the attitudes, perspectives, and knowledge of future Pakistani female pharmacy students concerning BSE through their participation in screening programs and breast control measures. The study also aims to evaluate changes in their knowledge level, attitudes, and views regarding BSE and breast cancer following a specifically designed educational intervention. This project seeks to bridge the knowledge gap and empower medical professionals, policymakers, and the community at large to make well-informed decisions and collaborate to improve women’s breast health outcomes.

## Materials and methods

### Study site and study design

A pre-post quasi-experimental study was conducted from October to December 2023 with the aim of evaluating and improving knowledge, perceptions, and attitudes toward breast cancer among female pharmacy university students in Karachi, Pakistan. The participants were from the pharmacy departments of two different universities, one a private female university with morning and evening shifts and the other a co-educational public university.

### Inclusion and exclusion criteria

The study included women aged 18 years and above. Excluded from the study were women under 18, men, and individuals who declined to participate.

### Ethical considerations

The Jinnah University for Women Institutional Review Board granted ethical approval for the study (JUW/IERB/PHARM-ARA-007/2023), and permission to conduct the sessions was obtained from the deans of both universities. All students who participated in the study did so voluntarily, and their signed consent was obtained. The study’s objectives were thoroughly explained to each participant, and they were assured that their participation would remain anonymous, with no personal data collected. All information gathered was kept confidential and used exclusively for research purposes.

### Questionnaire validation

The content validity of both questionnaires was evaluated using Lawshe’s test and quantitative face validity through impact scores. Reliability and necessity were measured using the Content Validity Index (CVI) and Content Validity Ratio (CVR), with items rated by 10 experts. Items with a CVR value greater than 0.62 were deemed acceptable according to the Lawshe Table (15). Questionnaire relevance was evaluated using the CVI, where 10 experts rated items on a scale from 1 to 4.

The S-CVI/Ave scores were 0.914 for the pre-session and 0.9375 for the post-session questionnaires, with I-CVI values exceeding the minimum threshold of 0.78. The CVR values were 0.905 and 0.913, respectively, indicating good validity for the instruments (16, 17). Quantitative face validity was conducted with 20 participants using the item impact method, where values greater than 1.5 indicated the appropriateness of items (18). The results for face validity showed that all items in the pre- and post-survey achieved impact scores higher than 1.5, indicating the appropriateness of all items.

### Data collection and procedure

#### Baseline questionnaire administration

The study was conducted on 1,200 female pharmacy students recruited from two different universities. The consented participants were provided with a baseline questionnaire that had been previously validated, pilot-tested, and self-administered. The questionnaires were adapted from previously published studies and expert opinions (19–22).

The baseline questionnaire was divided into three sections:

*Section 1*: This section includes information regarding the participant’s socio-demographic information, including age, marital status, history of breast cancer in family/relatives and friends, their past breast cancer practices, and attendance at breast cancer sessions. Their names and academic years were also obtained to ensure follow-up for the post-session survey.
*Section 2*: This section consists of two main parts. Part one evaluated participants’ general perceptions and knowledge regarding breast cancer through six questions. Part two focused on attitudes toward breast cancer, which was assessed through four questions.
*Section 3*: This section was divided into three parts. Part one included 10 risk variables, part two included eight variables regarding symptoms, and part three comprised four questions to evaluate the participants’ understanding of BSE techniques. This included their familiarity with screening methods for identifying breast cancer, the frequency (e.g., “weekly,” “monthly”), and timing of BSE (e.g., “before menstruation,” “any time”), as well as the accurate age for each screening method.

### Educational intervention

Awareness workshops were conducted over a period of 6 months, consisting of a 45-min awareness session, followed by a 7 min short video tutorial. At the end of each session, an information leaflet was provided to each participant. Then, 2 weeks after the session, the target population was provided with post-session questionnaires to assess the impact of the educational training. The pre- and post-session surveys used in this research were based on those utilized in related earlier investigations (19, 20, 23–25).

### Post-session survey

The post-session form included only section two and three to assess changes in knowledge and perceptions following the educational intervention, as section one covered participants’ demographics and their past knowledge and practices.

**Figure 1:**
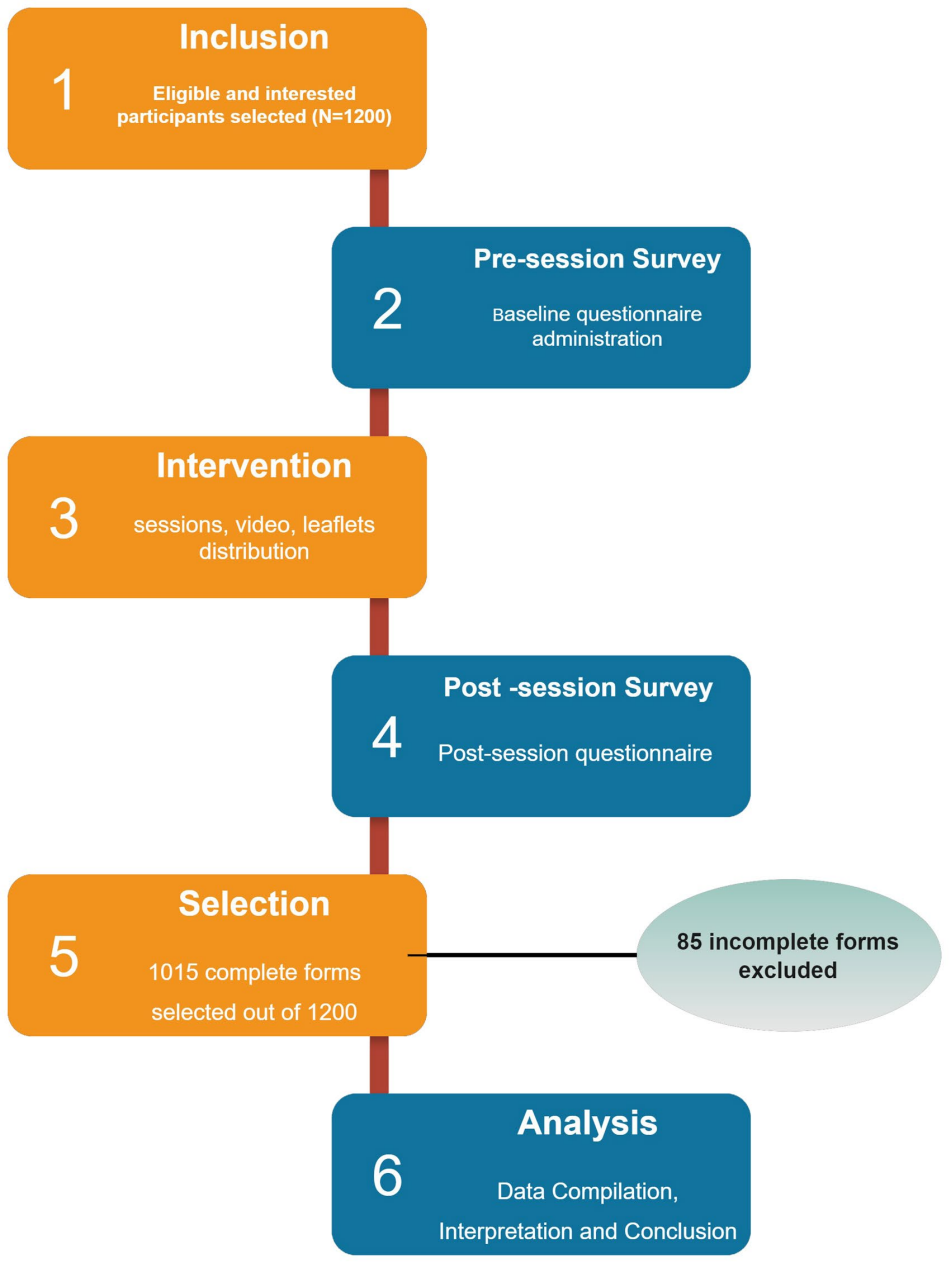
Workflow demonstration for conducting educational session.

Each participant was assigned a specific number during the pre-session, which was linked to their post-session form to ensure the correct pairing of both the pre- and post-questionnaires (Figure 1).

### Statistical workout

Following data collection, IBM SPSS statistics version 21 and Microsoft Office Excel were used to organize, code, and tabulate the data. Participants’ knowledge was categorized based on their scores in different domains: those scoring above 50% were classified as having adequate knowledge, while those scoring below 50% were classified as having inadequate knowledge. For instance, the participants who answered three or more questions correctly out of six in section two were considered knowledgeable. Pearson’s Chi-square test, dependent sample t-test, and ANCOVA (analysis of co-variance) were utilized to examine differences in knowledge before and after the session. Analysis was conducted using counts and percentages for categorical variables. SPSS version 21 was used for statistical analysis, and a *p-*value < 0.05 was considered statistically significant (20, 26).

## Results

### Participants demographics and past practices

After a thorough evaluation and the exclusion of incomplete questionnaires, out of 1,200 participants, 1,015 of them completed pre- and post-session questionnaires were selected for analysis. Table 1 shows that 83.9% (*n* = 852) of the students were aged 18–24 years, while 16.1% (*n* = 163) were aged 25–34 years. Among these students, 26.8% (*n* = 272) were married, and 73.2% (*n* = 743) were single. The participants were distributed across different academic years: 12.9% (n = 131) were first-year students, 11.8% (*n* = 120) were second-year students, with the majority being third-year students at 27.3% (*n* = 277), followed by final-year students at 24.3% (*n* = 247), and fourth-year students at 23.6% (*n* = 240).

**Table 1:** Participants’ demographics and past practices.

Demographic feature	% (*N*)
Age	
18–24 years	83.9% (*n* = 852)
25–34 years	16.1% (*n* = 163)
35 and above	0% (0)
Marital status	
Single	73.2% (*n* = 743)
Married	26.8% (*n* = 272)
Separated/divorced	0% (0)
Widow	0% (0)
Has anyone close to you ever been diagnosed with breast cancer?	
Family member	14.2% (*n* = 144)
Relative	21% (*n* = 213)
Friend	11.7% (*n* = 119)
No one	39.8% (*n* = 404)
Others	13.3% (*n* = 135)
Professional year	
1st year	12.9% (*n* = 131)
2nd year	11.82% (*n* = 120)
3rd year	27.29% (*n* = 277)
4th year	23.6% (*n* = 240)
5th year	24.33% (*n* = 247)
Have you attended any breast cancer awareness campaigns or initiatives?	
Yes	17% (*n* = 176)
No	83% (*n* = 839)
Do you perform BSE?	
Yes	39.3% (*n* = 399)
No	60.7% (*n* = 616)
If not, what is the main reason for not performing?	
Unaware of the need	24.2% (*n* = 246)
Do not have any breast-related issues	23.3% (*n* = 236)
Do not know how to examine it	13.2% (*n* = 134)

Regarding breast cancer history, 39.8% (*n* = 404) of the participants reported that no one in their social circle or family had been diagnosed with breast cancer, while 21% of the participants (*n* = 213) reported a history of breast cancer in their relatives, followed by 14.2% (*n* = 144) in their immediate family, 13.3% (*n* = 135) in others, and 11.7% (*n* = 119) in their friends. Only 17% of participants (*n* = 176) had attended breast cancer awareness campaigns or initiatives, while 83% of them (*n* = 839) had not. Among all participants, 39.3% of participants (n = 399) reported practicing breast self-exams regularly. However, 60.7% of participants (*n* = 616) did not practice breast self-exams regularly, with 24.2% of them (*n* = 246) stating they were unaware of the need, 23.3% of them (*n* = 236) believing they had no breast-related issues, and 13.2% of them (*n* = 134) indicating they were not familiar with breast self-exam techniques.

### Impact on knowledge against general perceptions towards breast cancer

As shown in Table 2, the initial perception of breast cancer revealed that less than 50% of the participants considered breast cancer a significant health issue and or believed that it was curable. After the educational intervention, these perceptions improved significantly, with 70 and 56% of participants acknowledging breast cancer as a significant health issue and curable, respectively. Similarly, the belief that early diagnosis influences treatment and prolongs life increased from 40 and 47.1% before the session to 75.9 and 65.3% afterward. The percentage of participants who recognized that every woman is at risk of developing breast cancer rose significantly from 43 to 79.3% post-session. Additionally, awareness that men can also develop breast cancer improved from 45% before the session to 72.3% afterward.

**Table 2:** General perceptions.

S. No.	Variables	Pre-session		Post-session		*p*-value
		Yes	No	Yes	No	
1.	Breast cancer is a significant health issue	37.6% (*n* = 382)	62.4% (*n* = 633)	70.0% (*n* = 710)	30.0% (*n* = 305)	0.005
2.	Breast cancer is curable	36.4% (*n* = 369)	63.6% (*n* = 646)	56% (*n* = 568)	44% (*n* = 447)	0.021
3.	Early diagnosis of breast cancer influences treatment	40.0% (*n* = 406)	60.0% (*n* = 609)	75.9% (*n* = 770)	24.1% (*n* = 245)	<0.001
4.	Early diagnosis of breast cancer guarantees prolonged life	47.1% (*n* = 478)	52.9% (*n* = 537)	65.3% (*n* = 663)	34.7% (*n* = 352)	0.006
5.	Every woman is at risk of developing breast cancer.	43% (*n* = 436)	57% (*n* = 579)	79.3% (*n* = 805)	20.7% (*n* = 210)	0.002
6.	Men can have breast cancer	45.0% (*n* = 457)	55.0% (*n* = 558)	72.3% (*n* = 734)	27.7% (*n* = 281)	0.002

### Impact on attitudes toward breast cancer

Table 3 summarizes the changes in attitudes toward breast cancer as a result of this session. Initially, 68.7 and 61.7% of participants believed that self-examination was unnecessary and that there was no need for breast examination in the absence of symptoms. After the session, these views shifted significantly, with only 39.4 and 29.8% holding these beliefs (*p* < 0.001 and *p* = 0.005, respectively). Additionally, 40.6 and 31.1% of participants initially expressed reluctance to discuss their breast health with family/friends and physicians, respectively. This reluctance decreased significantly after the session, with 68.2 and 58.4% of participants becoming more open to these discussions (*p*-value = 0.003 and 0.002, respectively).

**Table 3:** Attitudes toward breast cancer.

S. No	Variables	Pre-session		Post-session		*p*-value
		Yes	No	Yes	No	
1.	Self-examination is of no use and does not detect any abnormality	68.7% (*n* = 697)	31.3% (*n* = 318)	39.4% (*n* = 400)	60.6% (*n* = 615)	<0.001
2.	In the absence of symptoms, there is no need to examine your breasts	61.7% (*n* = 626)	38.3% (*n* = 389)	29.8% (*n* = 302)	70.2% (*n* = 713)	0.005
3.	Would you be open to discussing your breast health with your friends and family?	40.6% (*n* = 412)	59.4% (*n* = 603)	68.2% (*n* = 692)	31.8% (*n* = 323)	0.003
4.	Would you be open to discussing your breast health with your physician if you note any changes in your breasts?	31.1% (*n* = 316)	68.9% (*n* = 699)	58.4% (*n* = 593)	41.6% (*n* = 422)	0.002

### Impact on knowledge of breast cancer risk factors

Table 4 shows the participants’ knowledge of breast cancer risk factors before and after the intervention. Initially, less than 50% of the participants were aware of risk factors such as early menstruation (36.1%), late menopause (25.5%), having a first child at an older age (20.1%), post-menopausal hormone replacement therapy (23.2%), radiation exposure (30.3%), and no breastfeeding (46.6%). After the session, there was significant progress (*p* < 0.05), with knowledge increasing to 73.4, 74.9, 74.4, 89.8, and 67.0%, respectively. The smallest increase was observed in awareness of the no breastfeeding factor, which rose to 58.5% post-session.

**Table 4:** Risk factors awareness.

S. No	Variables	Pre-session % (*N*)		Post-session % (*N*)		*p*-value
		Yes	No	Yes	No	
1.	Family history of breast cancer	64.2% (*n* = 652)	35.8% (*n* = 363)	89.5% (*n* = 908)	10.5% (*n* = 107)	<0.001
2.	Early menstruation (<12 years)	36.1% (*n* = 366)	63.9% (*n* = 649)	73.4% (*n* = 745)	26.6% (*n* = 270)	<0.001
3.	Late menopause (>55 years)	25.5% (*n* = 259)	74.5% (*n* = 756)	74.9% (*n* = 760)	25.1% (*n* = 255)	0.021
4.	Having a first child at an older age (>30 years)	20.1% (*n* = 204)	79.9% (*n* = 811)	74.4% (*n* = 755)	25.6% (*n* = 260)	0.035
5.	Contraceptive use	50.3% (*n* = 511)	49.7% (*n* = 504)	51.2% (*n* = 520)	48.8% (*n* = 495)	0.979
6.	Post-menopausal hormone replacement therapy	23.2% (*n* = 235)	76.6% (*n* = 780)	89.8% (*n* = 911)	10.2% (*n* = 104)	0.015
7.	Alcohol/smoking	49.8% (*n* = 505)	50.2% (*n* = 510)	49.9% (*n* = 506)	50.1% (*n* = 509)	0.016
8.	Lack of physical activity	52.4% (*n* = 532)	47.6% (*n* = 438)	50.0% (*n* = 507)	50.0% (*n* = 508)	0.064
9.	Exposure to radiation	30.3% (*n* = 308)	69.7% (*n* = 707)	67.0% (*n* = 680)	33.0% (*n* = 335)	0.001
10.	No breastfeeding	46.6% (*n* = 473)	53.4% (*n* = 542)	58.5% (*n* = 594)	41.5% (*n* = 421)	<0.001

**Figure 2:**
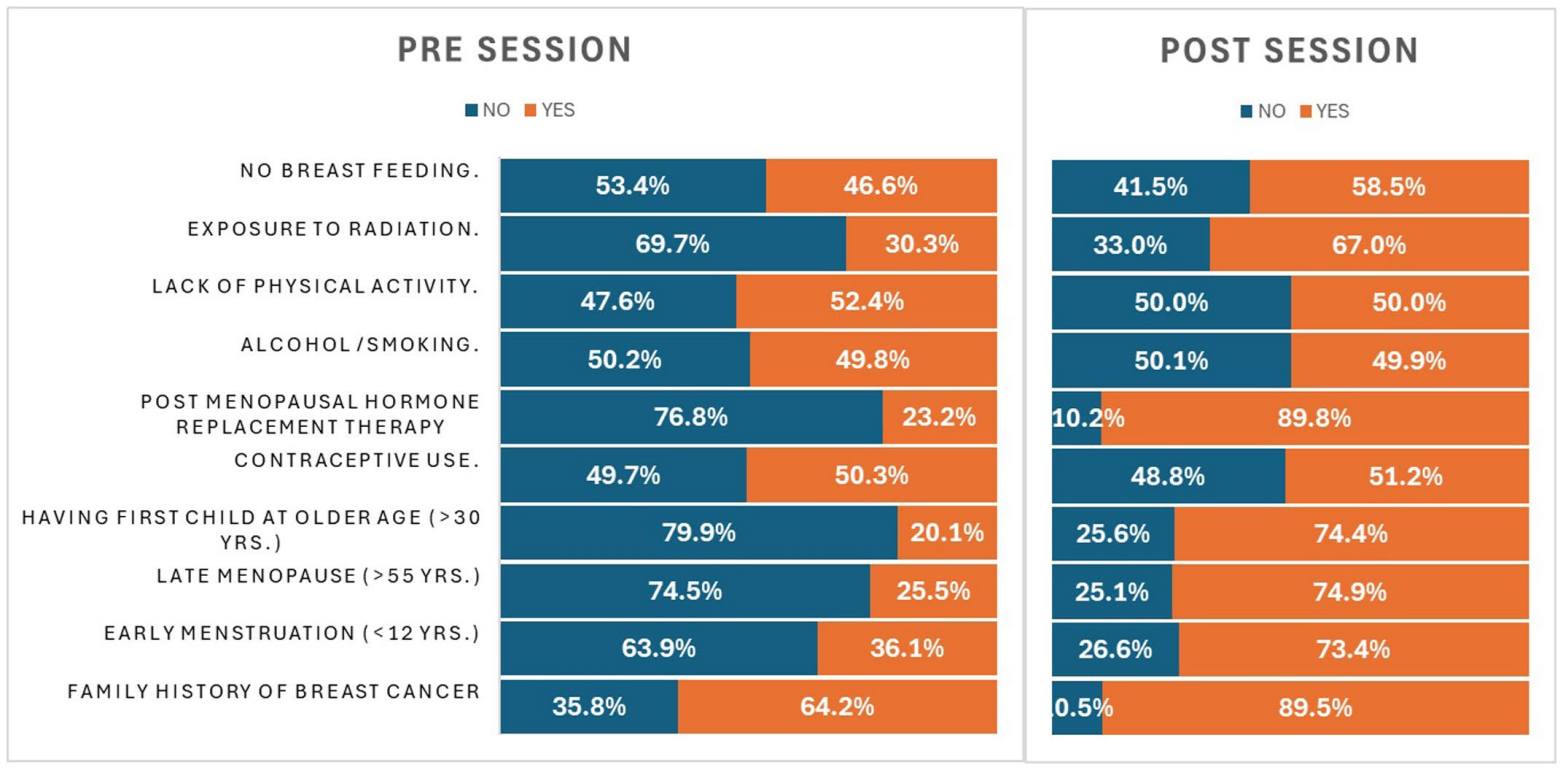
Graphical representation demonstrating change in knowledge on risk factors.

More than 50% of the participants already had knowledge of risk factors such as family history (64.2%) and lack of physical activity (52.4%). After the session, there was a significant increase in awareness of family history (89.5%), while a slight decline was noted in awareness of the lack of physical activity (50.0%). Regarding factors such as contraceptive use and alcohol/smoking, nearly 50% of the participants (50.3 and 49.8%) were well aware of these factors, and there was no significant change in their awareness levels after the session (51.2 and 49.9%). Figure 2 provides a graphical elucidation of this section.

### Impact on knowledge of breast cancer features

**Figure 3:**
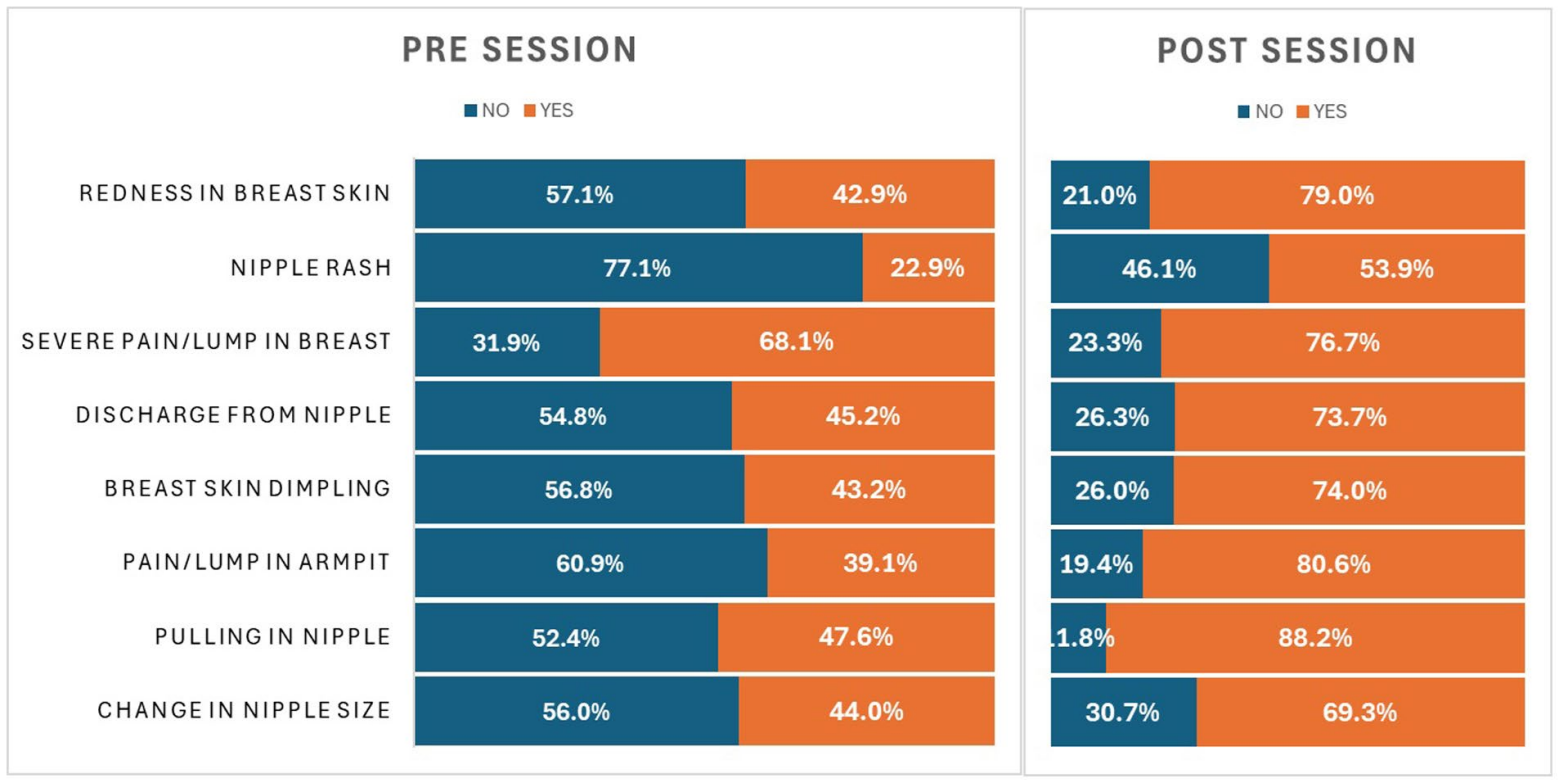
Graphical representation demonstrating change in knowledge on symptoms.

Table 5 reflects the impact on knowledge related to breast cancer signs/symptoms after the session. Our findings demonstrated that knowledge regarding changes in nipple size and nipple discharge was inadequate before the session (39.1 and 22.8%, respectively) but significantly improved post-session to 80.5 and 53.8% (i.e., <0.001). Knowledge of other variables such as pulling in nipples, pain/lump in the armpit, severe pain/lump in the breast, and nipple rash was also below 50% before the session (43.1, 45.2, 42.8, and 40.7%, respectively). After the session, knowledge increased to 73.9, 73.6, and 78.9% for these variables, except for nipple rash, which remained inadequate at 43.1% post-session. These improvements were statistically significant (*p* = 0.009, 0.020, 0.002, and 0.006, respectively). Knowledge regarding breast skin dimpling and redness was already adequate before the session (68.0 and 58.0%, respectively) but still showed a minor significant increase after the session (76.6 and 60.9%) with *p*-values of 0.034 and 0.002 (see Figure 3).

**Table 5:** Symptom awareness of breast cancer.

S. No	Variables	Pre-session % (*N*)		Post-session % (*N*)		*p*-value
		Yes	No	Yes	No	
1.	Change in nipple size	39.1% (*n* = 397)	60.8% (*n* = 618)	80.5% (*n* = 818)	19.4% (*n* = 197)	<0.001
2.	Pulling in the nipple	43.1% (*n* = 438)	56.8% (*n* = 577)	73.9% (*n* = 751)	26.0% (*n* = 264)	0.009
3.	Pain/lump in the armpit	45.2% (*n* = 459)	54.7% (*n* = 556)	73.6% (*n* = 748)	26.3% (*n* = 267)	0.020
4.	Breast skin dimpling	68.0% (*n* = 691)	31.9% (*n* = 324)	76.6% (*n* = 778)	23.3% (*n* = 237)	0.034
5.	Discharge from the nipple	22.8% (*n* = 232)	77.1% (*n* = 783)	53.8% (*n* = 547)	46.1% (*n* = 468)	<0.001
6.	Severe pain/lump in the breast	42.8% (*n* = 435)	57.1% (*n* = 580)	78.9% (*n* = 802)	21.0% (*n* = 213)	0.002
7.	Nipple rash	40.7% (*n* = 413)	59.3% (*n* = 602)	43.1% (*n* = 437)	56.9% (*n* = 578)	0.006
8.	Redness in breast skin	58.0% (*n* = 589)	42% (*n* = 426)	60.9% (*n* = 619)	39.0% (*n* = 396)	0.002

### Impact on breast cancer screening technique knowledge

Table 6 shows the impact on knowledge related to breast cancer screening routines, which was found to have a significant effect (*p* < 0.001) on almost all domains after the session. Before the session, less than 50% of the participants had knowledge regarding the frequency of BSE (46.0%), the correct time to perform them (29.3%), or the appropriate ages for mammography (38.3%), and CBE (33.9%). After the intervention, there was a significant improvement in awareness, with knowledge levels rising to 69.7, 72.7, 81.4, and 80.1%, respectively.

**Table 6:** Screening awareness of breast cancer.

S. No	Variables	Pre-session % (*N*)		Post-session % (*N*)		*p*-value
		Correct	In-correct	Correct	In-correct	
1.	Frequency of performing BSE	46.0% (*n* = 467)	54.0% (*n* = 548)	69.7% (*n* = 707)	30.3% (*n* = 308)	<0.001
2.	BSE performing time	29.3% (*n* = 297)	70.7% (*n* = 718)	72.7% (*n* = 738)	27.3% (*n* = 277)	<0.001
3.	The best age for a mammography scan	38.3% (*n* = 389)	61.7% (*n* = 626)	81.4% (*n* = 826)	18.6% (*n* = 189)	<0.001
4.	The best age for a clinical breast exam	33.9% (*n* = 344)	66.1% (*n* = 671)	80.1% (*n* = 813)	19.9% (*n* = 202)	<0.001

### Impact on knowledge of professional years

Table 7 shows the impact of the educational intervention on knowledge across different professional years. Before the session, students from each year displayed overall inadequate knowledge about breast cancer, with 11.5% of first-year students, 12.5% of second-year students, 17% of third-year students, 13.3% of fourth-year students, and 12.1% of final-year students having sufficient knowledge. After the session, knowledge significantly improved across all years, with over 90% of students demonstrating adequate understanding, reflecting the success of the intervention.

**Table 7:** Impact on knowledge of professional years.

S. No	Professional year	Pre-session % (*N*)		Post-session % (*N*)		*p*-value
		Adequate	In-adequate	Adequate	In-adequate	
1.	1st year	11.5% (15)	88.5% (116)	98.5% (129)	1.5% (2)	
2.	2nd year	12.5% (15)	87.5% (105)	97.5% (117)	2.5% (3)	<0.001
3.	3rd year	17% (47)	83.0% (230)	99.3% (275)	0.7% (2)	
4.	4th year	13.3% (32)	86.7% (208)	99.6% (239)	0.4% (1)	
5.	5th year	12.1% (30)	87.9% (217)	98.8% (244)	1.2% (3)	

### Complete case analysis of pre-post interventional studies

**Figure 4:**
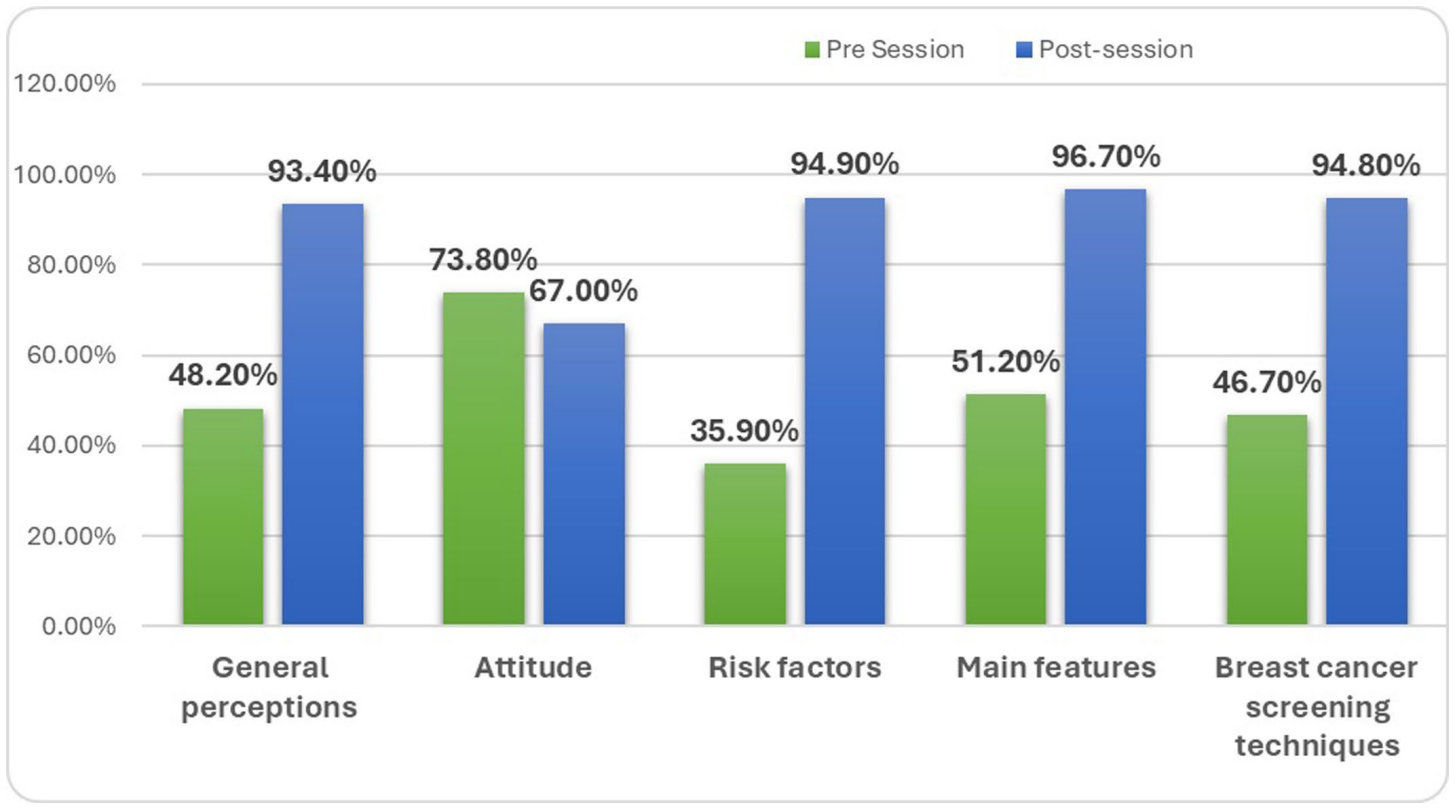
Graphical representation demonstrating overall impact on knowledge.

The designed awareness session was effective in significantly increasing overall awareness of breast cancer, altering public perceptions, and improving knowledge and attitudes. Table 8 and Figure 4 illustrate the overall increase in awareness across various domains, expressed in percentages. Knowledge of risk factors and screening methods showed a significant increase, rising from 35.9 and 46.7% and from 94.9 and 94.8%, respectively, with *p*-values of 0.010 for risk factors and < 0.001 for screening techniques. The most substantial impact was observed in the knowledge of breast cancer features, which increased from 51.2 to 96.7%, with a significant *p-*value of 0.022.

**Table 8:** Overall awareness of breast cancer in terms of percentages.

S. No	Variables	Pre-session % (*N*)		Post-session % (*N*)	
		Adequate	Inadequate	Adequate	Inadequate
1.	Overall impact of general perceptions	48.2% (*n* = 489)	51.8% (*n* = 526)	93.4% (*n* = 948)	6.6% (*n* = 67)
2.	Overall changes in the attitude	73.8% (*n* = 749)	26.2% (*n* = 266)	67.0% (*n* = 687)	32.3% (*n* = 328)
3.	Overall awareness of risk factors	35.9% (*n* = 364)	64.1% (*n* = 651)	94.9% (*n* = 963)	5.1% (*n* = 52)
4.	Overall awareness of main features	51.2% (*n* = 520)	48.8% (*n* = 495)	96.7% (*n* = 981)	3.3% (*n* = 34)
5.	Overall awareness of breast cancer screening techniques	46.7% (*n* = 474)	53.3% (*n* = 541)	94.8% (*n* = 962)	5.2% (*n* = 53)

General perceptions of breast cancer improved from 48.2 to 93.4%, with a significant relationship (*p*-value 0.019). However, there was a significant decline in certain attitudes, with the percentage changing from 73.8 to 67.0% (*p*-value 0.009).

This decline aligns with the findings discussed earlier in Table 3, where 68.7 and 61.7% of the participants initially considered self-examination unnecessary or believed that there was no need for breast examination in the absence of symptoms. After the session, these views shifted to 39.4 and 29.8%, respectively. Table 9 presents the overall interventional effect, showing a significant shift in mean scores from 13.5 ± 2.68 to 21.92 ± 2.49, with an overall effect size of 8.42.

**Table 9:** Complete case findings of pre-post interventional studies.

Domain	Total scores	PRE		Post		Difference	*p*-value
		Mean	Standard deviation	Mean	Standard deviation		
General perception	6	2.49	1.22	4.19	1.11	1.70	0.019
Attitude	4	2.02	0.89	1.96	0.95	−0.06	0.009
Risk factor	10	3.99	1.46	6.78	1.41	2.80	0.010
Symptoms	8	3.53	1.38	5.95	1.20	2.42	0.022
Screening	4	1.47	0.98	3.04	0.87	1.56	<0.001
Overall interventional effect	32	13.50	2.68	21.92	2.49	8.42	<0.001

## Discussion

Given the increasing prevalence of breast cancer in recent years in Pakistan (27), this study was designed as an effort to assess the basic level of education regarding breast cancer among pharmacy students and to enhance their awareness to better guide future generations. The study was implemented in three phases. Initially, a pre-session questionnaire was administered to evaluate the participants’ basic knowledge of breast cancer, including its features, risk factors, screening techniques, and general perceptions and attitudes toward breast cancer. Following this, an educational intervention was conducted, consisting of a 45-min session, a 7-min brief video, and precise educational leaflets. After 2 weeks, the same students were asked to complete a post-session questionnaire. The pre-test and educational intervention were conducted on the same day.

After analysis, it was determined that the overall knowledge of these students increased as a result of the planned educational intervention. Awareness improved across all aspects of breast cancer, including knowledge of its symptoms, risk factors, screening methods, and general perceptions, with positive changes in attitudes as well. The purpose of involving medical students in this session was to assess their baseline knowledge levels and to enhance their understanding of medical terms. The results of this study confirm that baseline knowledge regarding breast cancer was inadequate but could be significantly improved through the introduction of such educational sessions on a yearly basis.

Overall awareness of risk factors and screening methods increased significantly, reaching approximately 94.9 and 94.8%, respectively. The most significant impact was seen in the increase in knowledge of breast cancer features, which rose from 51.2 to 96.7%. General perceptions also improved, shifting from 48.2 to 93.4%, with a significant relationship. A closer examination of the results revealed that overall knowledge improved to over 90%, with a significant shift in the overall interventional effect, as indicated by the mean scores increasing from 13.5 ± 2.68 to 21.92 ± 2.49. This confirms the success of the study.

Available literature confirms that studies like this, conducted in different schools, universities, and other settings to promote breast cancer awareness, have a positive impact on increasing awareness (28, 29). For instance, a study conducted on a group of Nigerian adolescents used peer education in a pre-post interventional study to raise breast cancer awareness. This study found a significant improvement in baseline education after the educational intervention and demonstrated that such an approach could be cost-effective and easily implemented with limited resources (21). Similarly, a study conducted at a female university in Bangladesh successfully enhanced knowledge and awareness about breast cancer and BSE practices (28).

In Malaysia, a randomized controlled trial was conducted among Yemeni female school teachers, involving both control and intervention groups. The intervention group received a 1-day educational session, including a 90-min presentation on breast cancer screening, while the control group was only provided with similar educational material. The study aimed to assess the effectiveness of the educational intervention at different time intervals, with the goal of educating teachers so that they could, in turn, educate younger generations about breast cancer awareness (30).

In another study conducted in Bangladesh, a hospital-based survey among women revealed that a lack of knowledge and awareness programs, sociocultural norms, disease-related fear, and shyness were major barriers that prevented women from consulting or communicating their condition to anyone, including physicians (31).

However, further research showed a rise in the recognition of early-stage breast cancer among nurses and other healthcare professionals who had received CBE training. Additionally, CBE was shown to reduce the percentage of late-stage breast cancer recurrence by 50% (32).

In previous years, the American Cancer Society recommended that women begin performing BSE in their 20s and continue them regularly throughout their lives. However, these recommendations have evolved over time. The most recent guidelines, updated in 2015, emphasize the importance of breast awareness rather than adhering to a strict regimen or schedule for formal BSE (33). These discussions about breast health and its significance must continue throughout a woman’s life, beginning in adolescence and continuing into adulthood.

This study has several strengths. First, the questionnaire was validated by experts and oncologists. Every session and the completion of both questionnaires were conducted under supervision. Confidentiality was a top priority and was maintained throughout all stages of the intervention. However, there are also some limitations to this study. The results can be generalized to female pharmacy students but may not apply to all women or represent all healthcare professionals. Additionally, a follow-up session regarding breast self-exam practices could not be conducted due to time constraints.

## Conclusion

Organizing breast cancer awareness sessions is particularly important in lower-middle-income countries, where access to healthcare and knowledge about the disease may be limited. These sessions serve as critical platforms for educating individuals about the warning signs and risk factors of breast cancer. By providing education on self-examinations, the importance of routine screenings, and the need for prompt medical attention, these workshops empower women to take an active role in managing their health. Raising awareness facilitates early detection, which can be life-saving by ensuring that those affected receive appropriate care and treatment in a timely manner. Such awareness initiatives have the potential to significantly improve healthcare outcomes, reduce mortality rates, and enhance the overall wellbeing of communities in lower-middle-income countries by fostering a culture of education and proactive health management.

## Data Availability

The original contributions presented in the study are included in the article/supplementary material, further inquiries can be directed to the corresponding author.
